# “We Want to Eat and be Healthy just like Everybody Else:” How Social Infrastructures Affect Nutrition Equity in a Racialized Urban Community in the United States

**DOI:** 10.1016/j.cdnut.2024.102106

**Published:** 2024-02-21

**Authors:** Gabby Headrick, Kiera Abdul, Shireen Guru, Allison DeHonney, Alyssa J. Moran, Pamela J. Surkan, Samina Raja, Yeeli Mui

**Affiliations:** 1 The Milken Institute School of Public Health, George Washington University, Washington, DC, United States; 2 The Johns Hopkins Bloomberg School of Public Health, Johns Hopkins University, Baltimore, MD, United States; 3 The University at Buffalo School of Architecture and Planning, The University at Buffalo State University of New York, Buffalo, NY, United States; 4 Urban Fruits & Veggies, Buffalo, NY, United States

**Keywords:** food security, equity, SNAP, social networks, urban, social infrastructures

## Abstract

**Background:**

Food security and nutrition equity, 2 social determinants of health, are impacted by the coronavirus disease 2019 (COVID-19) pandemic and the racialization of urban communities. Few studies to date have examined how the use of social infrastructures in the United States during COVID-19 affected the ability to achieve food security and nutrition equity.

**Objectives:**

To describe how the use of social infrastructures impacts food security and nutrition equity in a majority Black and urban community in the United States.

**Methods:**

Semistructured in-depth interviews were conducted with 40 low-income, urban, and predominately Black people living in Buffalo, New York in May–July 2022.

A thematic analysis using a phronetic iterative approach informed by the Social Ecological Model, Walsh’s Family Resilience Framework, and a framework focused on the advancement of nutrition equity.

**Results:**

We identified 9 themes mapped across 3 interrelated domains that impact nutrition equity, including *1*) meeting food needs with dignity, *2*) supply and demand for fresh and healthy foods, and *3*) community empowerment and food sovereignty. We found that people used coping strategies, such as food budgeting and cooking skills, paired with different social infrastructures to meet food needs. People commonly used the Supplemental Nutrition Assistance Program and food pantries to meet food needs over receiving support from family members or friends outside of the household. Poverty, challenges accessing and affording healthy food, and the inability to reciprocate support to others undermined the advancement of nutrition equity despite social infrastructures being available for use. Historical and ongoing acts of disempowerment and disinvestment also hindered the advancement of nutrition equity.

**Conclusions:**

Sustained, community-led investment is needed to address structural inequities preventing the advancement of nutrition equity. Social infrastructures should be expanded to inclusively support low-income populations, so wealth generation is possible to address the root cause of food insecurity.

## Introduction

The COVID-19 pandemic magnified inequities in food insecurity in the United States [1]. In 2021, an estimated 10.5%, or 13.5 million United States households, experienced food insecurity, meaning that at some point during the year, the household lacked enough safe, quality, and desirable food for all members of the household because of a lack of resources to get food in socially appropriate ways [1]. The burden of food insecurity has remained higher among Black, Indigenous, and people of color (BIPOC) populations over the past decades [1]. In 2021, 19.8% of Black households and 16.2% of Latinx households experienced food insecurity compared with 7% of white households [1]. Furthermore, 24.3% of single, female-headed households and 31% of households living below 130% of the federal poverty level experienced food insecurity in 2021 [1]. Although national annual data suggest that food insecurity prevalence did not rise between 2020 and 2021, some surveillance and longitudinal data collected during the early months of the COVID-19 pandemic revealed that the economic and social hardships experienced may have contributed to worsening food insecurity disparities [[2], [3], [4]]. For example, a longitudinal study of Black residents living in Pittsburgh, Pennsylvania, found that food insecurity increased by nearly 80% in May of 2020 compared with 2018 [4].

**FIGURE 1:**
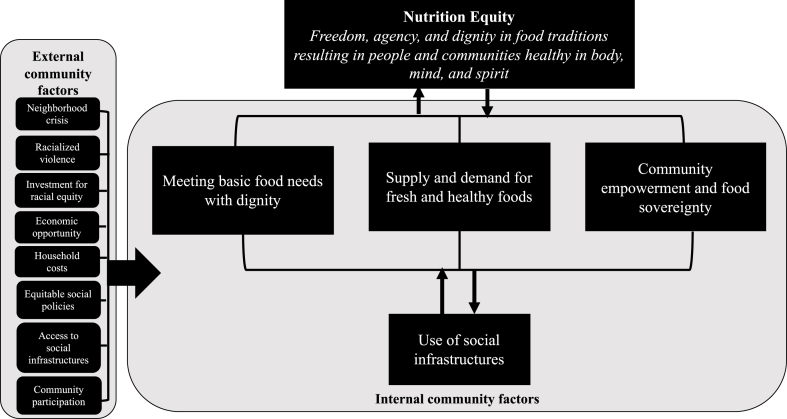
An adapted framework^1^ describing the advancement of nutrition equity in a racialized urban community. ^1^Adapted with permission from Freedman et al. [5].

In the United States, disinvestment in the form of historical and ongoing practices of segregation and discriminatory policies has created unequal access to healthy and culturally preferred foods in BIPOC communities [5]. Today, the resulting structural inequality is at the root of racial disparities in food insecurity and continues to undermine the fundamental human right of access to nutritious and culturally preferred foods, food sovereignty, and ultimately, the right to health [[5], [6], [7], [8], [9], [10]]. Thus, this study draws from a framework developed by Freedman et al. [5] that focuses on reducing disparities in food insecurity by advancing nutrition equity in racialized urban communities (Figure 1) [5]. Nutrition equity is defined as the “freedom, agency, and dignity in food traditions resulting in people and communities healthy in body, mind, and spirit” [5]. Freedman et al. [5] argue that BIPOC communities must be empowered both politically (for example, voter participation) and economically (for example, having fair access to land to grow food), must have a supply and access to healthy foods (for example, full-service grocery stores with affordable, fresh, and culturally preferred foods), and must be able to meet food needs with dignity (for example, using social benefit programs and charitable food sources without stigmatization) to advance nutrition equity [5]. The ability of these 3 domains to converge to promote nutrition equity is dependent on availability and utilization of resources (that is, social infrastructures), as well as external household, community, and sociopolitical contexts in which these resources exist [5].

To cope with food insecurity, people use different strategies and resources [[11], [12], [13], [14]], which we describe as social infrastructures [15,16]. We draw our conceptualization of social infrastructures from the United States Surgeon General [16] to include the programs, policies, physical environments, and relationships that foster vital social connections for people to gain resources (economic, emotional, material, etc.) to meet their human needs. One social infrastructure is the USDA’s Supplemental Nutrition Assistance Program (SNAP), which provides cash-like benefits for eligible low-income families to purchase groceries each month [17,18]. SNAP is the largest federal nutrition program, reaching over 40 million people 2022 [14]. Participation in SNAP grew dramatically during the COVID-19 pandemic as Congress passed legislation allowing states to temporarily modify enrollment processes and increase household benefits for the duration of the public health emergency [14,16]. The temporary benefit increase was the largest in the program’s history, with all households receiving the maximum monthly benefit amount for their household size, or an additional $95 in benefits per month (whichever was a greater increase) [19]. Although evidence of the impact of this unprecedented expansion of SNAP is currently limited, early analyses suggest the expansion of SNAP and other federal nutrition assistance benefits protected many households from falling deeper or into experiences of food insecurity [1,20,21]. Still, limited evidence exists describing how the use of SNAP promotes or undermines the pursuit of nutrition equity among BIPOC communities. This is imperative to investigate because the country continues to navigate the lasting impacts of the COVID-19 pandemic—particularly in racialized urban areas that have suffered a disproportionate burden of food insecurity [1].

Beyond SNAP, people also rely on family members and friends (that is, social networks), community centers, food pantries, food growing spaces, and mutual aid organizations to meet their food needs [22]. Evidence describing the relationship between the use of social networks and household food insecurity is inconsistent [23]. Some cross-sectional and qualitative studies suggest having people, specifically family members, to rely on in times of need can help mitigate food hardship by sharing financial resources and food [24]. Other descriptive studies suggest that social networks are not protective against food insecurity, and can even contribute to experiences of food hardship because of the need to provide food support to others when resources are limited [23,25,26]. Charitable food sources, including meal sites, food pantries, and mutual aid organizations, are commonly used by people experiencing food insecurity as a reactive strategy to try to meet food needs because of insufficient financial resources [27,28]. For example, during the pandemic, food available through the charitable food system dramatically increased, but the overall impact on food security and nutrition equity in a community is not clear [29]. In addition, how people used, or did not use, their social networks to meet food needs through the COVID-19 pandemic and national public health emergency is not understood.

The use of social infrastructures in the United States during COVID-19 and the effect on the ability to achieve food security and nutrition equity in a racialized urban community is understudied. To further illuminate the effect of social infrastructures, we conducted in-depth interviews with predominately Black, urban, and low-income households to describe *1*) how the use of social infrastructures affects food security and *2*) how this impacted the advancement of nutrition equity in a United States racialized urban community.

## Methods

### Setting

This research focused on 7 low-income neighborhoods in Buffalo, New York, and was conducted between May 2022 and July 2022. Buffalo is a rustbelt city and one of the most racially segregated urban cores in the United States [30]. Estimates suggested 73% of the population identified as Black in the study area compared with 36% in the entire city at the time of this research project [31]. Furthermore, the median household income in the study area was $10,000 lower than the rest of the city, resulting in nearly 40% of residents living in poverty and 35% of the population receiving and using SNAP [31].

It is important to situate this study in the current sociopolitical context of Buffalo. On May 14, 2022, a tragic mass shooting motivated by white supremacy and racism against Black people injured 13 people and took the lives of 10 [32]. This massacre occurred in the only supermarket in the study area at the beginning of our data collection. This violence remains a traumatic event for the people of Buffalo, specifically Black Buffalo. This massacre is recent and painful evidence of the racialization of the food system in Buffalo, which is the setting of this study.

### Study design, participants, and recruitment

#### Parent study and household survey

This research is nested within a participatory-action research project called “Growing Food Policy from the Ground Up” (GFPGU), which is focused both in Buffalo, New York, and Minneapolis, Minnesota. The research team is a collaboration between investigators at the University at Buffalo State University of New York, the University of Minnesota, the Johns Hopkins Bloomberg School of Public Health, and community investigators from organizations in both cities (Appetite for Change, Massachusetts Avenue Project, Food for the Spirit, and Urban Fruits & Veggies). The overarching goal of GFPGU is to strengthen and leverage the power of social networks and community, specifically among BIPOC residents, to create resident-imagined and led transformative food policy and system changes. One component of this multidisciplinary project was a cross-sectional household survey that described food security, health, social networks, and food acquisition behaviors among residents in the 7 focus neighborhoods in Buffalo. To be eligible to participate in the household survey, participants met the following inclusion criteria: *1*) 18 y or older, *2*) lived in 1 of the 7 focus neighborhoods, and (*3*) identified as the primary food shopper for their household. The research team conducted the household survey over the phone between August 2021 and January 2022 with a total of 258 people. At the end of the survey, participants consented or did not consent to be recontacted for involvement in future research studies.

#### Eligibility and recontact

After the completion of the household survey data collection, eligible participants were recontacted based on the following inclusion criteria: *1*) consented to be recontacted, *2*) current residence in 1 of the 7 focus neighborhoods, and *3*) an income level below ∼200% of the federal poverty level or participation in SNAP within the last year reported at the time of the survey. In total, 111 participants were eligible for this study. We aimed to describe differences between people experiencing food security and food insecurity and used a criterion-based sampling strategy to ensure theoretical saturation across the 2 groups. The list of eligible participants was segmented into those reporting food insecurity (*n* = 57, 51%) and those reporting food security (*n* = 54, 49%) at the time of the survey (additional characteristics of eligible participants are reported in Supplemental Table 1). Food security was measured using the USDA Six-item Short Form Food Security Survey Module [33]. Each list was randomized with an equal number of people experiencing food security and food insecurity being allocated to 2 trained data collectors (1 student investigator and 1 graduate-level research assistant, both trained in qualitative methods).

#### Recruitment and screening procedures

The data collectors contacted eligible participants for a total of 3 attempts via the phone. On successful contact, a screener was completed with the participant to confirm eligibility, document the current SNAP receipt status, and assess the current risk of food insecurity. The experience of food insecurity was reassessed at the time of the screener because the experience of food insecurity is not static [1]. We used the Hunger Vital Sign 2-item screener to determine food insecurity risk, which is a validated food insecurity screening tool that reduces response burden [34]. The 2 questions assess both worry about food running out and food not lasting before having economic resources to purchase more food over the last 12 mo [34]. Households were classified as at risk of food insecurity if they answered affirmatively (often true or sometimes true compared with never true) to either or both of the questions [34]. In total, the data collectors recruited 40 people through phone calls and completion of the screener, which was the anticipated number of people to recruit for theoretical saturation [35,36]. We estimated a needed sample size of 40 for data saturation given the scope of our topic of interest along with the depth and richness of information we anticipated to receive from each person included in our sample [35,36]. Past involvement in the household surveys and coinvestigators being trusted community leaders from Buffalo promoted rapport between participants and data collectors, providing rich data quality [35].

In total, 30 people were classified as at risk of food insecurity and 10 were classified as not at risk of food insecurity (food secure) at the time of the interview. Additional household characteristics (racial and ethnic identity, gender identity, household income, homeownership, vehicle ownership, education level, employment status, use of SNAP in the last year, use of other social benefit programs in the last year, use of charitable food support in the last 30 d, and household size) were drawn and summarized from data previously collected during participants’ household surveys.

### Data collection

#### Interview guide

We used a semistructured interview guide informed by Walsh’s Family Resilience Framework and the Social Ecological Model for data collection [37,38]. The Family Resilience Framework is grounded in a household’s strengths and seeks to understand the strategies and processes used to repair and grow in the face of hardship, emphasizing the assets of a family compared with their weaknesses [38,39]. The Social Ecological Model comprises 5 levels of factors that interact to influence the given outcome or behavior of interest [37]. These 5 levels include *1*) intrapersonal factors, *2*) interpersonal factors, *3*) institutional factors, *4*) community factors, and *5*) public policy factors. Given that households cope with food insecurity through a dynamic interplay of accessing and using various social infrastructures, the use of these frameworks aided in question development to ensure we gained an understanding of how households use these infrastructures across different physical and social spaces of their lives.

We asked a series of open-ended questions (Supplemental Table 2) that were sorted into 4 topic areas that focused on understanding a household’s process of using social infrastructures to meet household food needs. The topic areas included *1*) where people get food and food spending habits; *2*) views and experiences receiving and using SNAP benefits; *3*) use of support from family members, friends, and charitable food sources; and *4*) recommendations to be able to achieve food security. The guide was reviewed by the larger research team, which included content experts in qualitative methods, SNAP, food systems planning, health equity, and a community investigator from the GFPGU research team. Over the span of data collection, the guide was iteratively revised based on emerging themes and topics discussed in previous interviews. Revisions were made through weekly discussions with both data collectors and the senior author. In addition, revisions were made through consultation with community coinvestigators to reflect ongoing events in the community given the interviews occurred alongside community grieving and healing from the May 14 massacre.

#### Semistructured interviews

Two data collectors trained in qualitative methods and in-depth interviewing methodology completed all interviews over the phone following the informed verbal consent of the participant. Interviews lasted 45 min on average and occurred during an agreed upon and scheduled time established at the point of the screening interview. All participants, except 1, granted permission for the interview to be audio-recorded. The interview in which the audio recording was refused resulted in detailed note-taking that was used for coding and thematic analysis. All participants were given a $25 Visa gift card as compensation for their time. In addition, given the high prevalence of food insecurity in our sample and the interviews taking place in the aftermath of the May 14 massacre, all participants were offered immediate food and mental health services that were organized and provided by Seeding Justice, a coalition of Black-led community organizations led by Black Love Resists in the Rust [32]. The research study was reviewed and approved by the Institutional Review Board of the Johns Hopkins Bloomberg School of Public Health.

### Data management and analysis

#### Transcription and coding

All audio recordings were transcribed verbatim by a professional transcription company. The student investigator and research assistant (hereafter collectively referred to as the research team) deidentified and checked all transcripts for accuracy by listening to the audio recording while reading the transcripts line by line. To organize the data, the student investigator developed a preliminary codebook through line-by-line close reading and inductive coding of 5 transcripts. The codebook was discussed with the research assistant and pilot-tested by both the student investigator and research assistant on 4 transcripts not used in codebook development to begin to establish coder agreement. The research team met to discuss code use and definitions for the 4 transcripts used and modified the codebook based on the discussion. The research team continued to double-code pilot transcripts until no differences in code use or interpretation were visually or conceptually detected when comparing the coding across transcripts between research team members. In total, 8 transcripts (20%) were double-coded and discussed line by line to establish intercoder agreement over a 2-wk period. The final codebook included 76 codes organized across 4 code groups that were informed by the Social Ecological Model. All transcripts, including those used in codebook development and intercoder agreement, were divided between the 2 research team members with the research assistant independently coding 14 transcripts and the student investigator independently coding 26 transcripts. The research team coded in ATLAS.ti, meeting weekly for 4 wk to discuss the coding process (that is, progress, resolution of any coding question, etc.) and reflect on emerging themes.

#### Thematic analysis

After the organization of the data through coding procedures, the student investigator used a phronetic iterative approach to guide the thematic analysis [40]. The phronetic iterative approach uses inductive and deductive coding methods over 2 cycles of coding to organize the data and identify emergent themes based on the inductive codes derived from the data and prior literature to shape and inform themes reported [40]. In the first cycle of coding, the student investigator used the codebook developed (primary-cycle codes). Emerging themes from primary-cycle coding were discussed with the research assistant and the senior author/co-principal investigator of the GFPGU project team. In the second cycle of coding, the student investigator used an adapted version of Freedman et al.’s [5] framework (Figure 1) [5] for achieving nutrition equity in racialized urban communities to collapse codes to address the research questions. The student investigator summarized themes in memos and consolidated the themes into 3 domains informed by Freedman et al.’s [5] framework.

#### Reflexivity in the research process

The student investigator (and first author) is a white female living in an urban center outside of Buffalo, NY, who led this research while studying for her PhD in public health nutrition. Given this positionality to the research process, this lead investigator worked with members of the GFPGU research team in Buffalo, NY, to gain the needed community context. Throughout the process, this investigator wrote memos reflecting on her role in the research, acknowledging and bracketing assumptions, and documenting additional questions that emerged. To ensure rigor in the research process, themes were shared with the larger GFPGU research team, including community leaders from Buffalo, to member-check the analysis and ensure that the themes summarized reflected the reality of those living and working within the study area. Themes were confirmed from this process as valid and then finalized.

## Results

### Household characteristics

A total of 40 people living in the 7 focus neighborhoods in Buffalo, NY, completed in-depth interviews. The majority identified as at risk for household food insecurity (*n* = 30; 75%), as Black residents of Buffalo (*n =* 35, 88%), and as female (*n =* 28, 70%) (Table 1) [34]. The mean age of the sample was 53.3 y. The majority (*n =* 34, 85%) reported a household income under $25,000, rented their home (*n =* 35, 88%), and did not own a vehicle (*n =* 23, 58%). Over half of people (*n =* 26, 65%) reported being unemployed. Most people interviewed received SNAP within the last 12 mo (*n =* 34, 85%), and under half previously reported using charitable food sources (*n =* 18, 45%). Only one-third of the people interviewed lived with children in the household, and the average household size in the sample was 2.5 people.

**TABLE 1:** Characteristics of people living in Buffalo, New York, in the study sample (*n* = 40)

Characteristic	Overall		At risk for food insecurity^1^		Food secure	
	*n* = 40	100%	*n* = 30	75%	n = 10	25%
Demographics						
Race/ethnicity, *n* (%)						
Black alone	35	88	25	83	10	100
White alone	3	8	3	10	0	0
Other race/ethnicity alone^2^	2	5	2	7	0	0
Gender identity, *n* (%)						
Female	28	70	22	73	6	60
Male	12	30	8	27	4	40
Mean age, y (SD)	53.3	12.4	51.5	2.4	58.9	2.8
Socioeconomics						
Household income, *n* (%)						
<$15,000	19	48	14	47	5	50
$15,000–$24,999	15	38	12	40	3	30
$25,000–$34,999	4	10	3	10	1	10
$35,000–$49,999	2	5	1	3	1	10
Homeownership, *n* (%)						
Rent	35	88	28	93	7	70
Own	5	13	2	7	3	30
Vehicle ownership, *n* (%)						
0 vehicles	23	58	19	63	4	40
≥1 vehicle	17	43	11	37	6	60
Education level, *n* (%)						
<High school	3	8	3	10	0	0
High school graduate	12	30	8	27	5	50
Some college	17	43	14	47	3	30
College degree^3^	7	18	5	17	2	20
Employment status, *n* (%)						
Full time	4	10	4	13	0	0
Part time	5	13	5	17	0	0
Unemployed	26	65	18	60	8	80
Retired	5	13	3	10	2	20
Receipt of SNAP in the last 12 months, *n* (%)						
Yes	34	85	26	87	8	80
No	6	15	4	13	2	20
Receipt of other social benefit programs, *n* (%)^4^						
Yes	2	5	2	7	0	0
No	38	95	28	93	10	100
Use of charitable food supports in the last 30 d, *n* (%)^5^						
Yes	18	45	15	50	3	30
No	22	55	15	50	7	70
Household composition						
Marital status, *n* (%)						
Married	5	13	3	10	8	80
Not married	35	88	27	90	2	20
Average household size, *n* (SD)	2.5	2.0	2.5	0.4	2.3	0.5
Any children in household, *n* (%)	13	33	10	33	3	30
Average number of children (*n* = 13), *n* (SD)	2.5	2.1	2.5	0.7	2.3	1.3

Abbreviation: SNAP, Supplemental Nutrition Assistance Program.

1 Risk for food insecurity assessed at the time of eligibility screener using the 2-item Hunger Vital Sign [34].

2 Includes Latinx, Asian, and Other race/ethnicity.

3 Includes Associate, Bachelors, and Graduate degrees.

4 Includes federal nutrition assistance programs, Farmers Market Incentives, Head Start, Temporary Assistance for Needing Families, Supplemental Security Income, Unemployment, Medicare, Medicaid, and Other Assistance.

5 Includes use of community food programs, food pantries, and faith-based organizations.

### Themes

We identified a total of 9 themes mapped to 3 domains of Freedman et al.’s [5] framework, which are defined in Table 2 [5]. The domains include *1*) meeting food needs with dignity (4 themes), *2*) supply and demand for fresh and healthy foods (2 themes), and *3*) community empowerment and food sovereignty (3 themes). Across all domains, we describe the processes of using (or not using) social infrastructures across different levels of society, informed by the Social Ecological Model. We describe differences observed between people identified as being at risk for food insecurity and those who identified as experiencing food security when differences in behavior or experiences were detected in the comparative thematic analysis. Differences are described when the majority of people experiencing food security (*n =* 5 or more people) described a reality or experience that was dissimilar from the reality or experience of the majority of people at risk for food insecurity (*n =* 15 or more people).

**TABLE 2:** Domains and themes describing the advancement of nutrition equity through the use of social infrastructures in a racialized urban community

Domain and definition^1^	Themes
1. Meeting food needs with dignity The ability to use social infrastructures to obtain food that promotes health and well-being absent of experiences of stigma, discrimination, and oppression.	1. Household budgeting, shopping, and cooking practices promote food security, but time and knowledge is needed to use these strategies
	2. The use of SNAP helps households meet their food needs, and the expansion of benefits during COVID-19 promoted food security
	3. Nutrition equity is undermined by negative experiences enrolling in, retaining, and using SNAP
	4. People navigate the complexities of the food system with resilience while trying to maintain independence from people outside their household
2. Supply and demand for fresh and healthy foods The availability and accessibility of foods within the immediate community that promote health, well-being and honor food culture.	1. Acute and chronic violence (i.e., interpersonal, and structural racism) against Black residents limits the supply of fresh foods despite the demand being present
	2. Charitable food sources supply relief but do not typically promote nutrition equity as the supply of nutritious foods is limited in many of these sources
3. Community empowerment and food sovereignty The investment of power, opportunity, and engagement into community through mechanisms that generate wealth in material, economic, political, and social resources for BIPOC people.	1. Community involvement through volunteerism and charity is present
	2. Immediate response and relief are necessary in times of crises but not sufficient to promote nutrition equity
	3. Attention and action must be given to the root causes of food insecurity

Abbreviations: BIPOC, Black, Indigenous, and people of color; SNAP, Supplemental Nutrition Assistance Program.

1 Adapted with permission from Freedman et al. [5].

### Domain 1: meeting food needs with dignity

#### Domain 1, theme 1: household budgeting, shopping, and cooking practices promote food security, but time and knowledge are needed to use these strategies

Universally, households described the importance of shopping for food with a budget in mind, particularly given inflated food costs at the time of the study. Many households shopped across multiple different grocery stores to find food items at the lowest price, but for some, this required multiple bus rides and took many hours to complete (Table 3, quote 1). Before shopping, some households described looking at weekly coupon flyers to inform where they would shop based on the lowest price. Only a few households said they shopped at places other than grocery stores and a meat market for food. For example, shopping at farmers markets in Buffalo was very rare among households interviewed, despite some farmer markets offering a nutrition incentive program for people who shopped with SNAP. Many people eligible for the incentive program reported not being aware of this benefit, highlighting a need to improve communication about resources available within the local food system.

**TABLE 3:** Illustrative quotes: meeting food needs with dignity

Quotation number	Quotation
1	“I’m an early bird. I’ll get up early, like 8:00 AM. I’ll take a bus. I go to Walmart first and after I go to Walmart I’ll… And I have a shopping cart, I’ll go to the Broadway market and after I leave the Broadway market and it’s a long day from busing us, I’ll go to Tops to see what’s on sale”—00DG_25
2	“My food always lasts because I am a good cook. Some people might think there is no food in the house, but I can throw something together with very little. For example, I like spaghetti. I might use like 6lbs of hamburger meat and then I freeze some. I might make a lot of chili, and then I freeze some. I make my food last. If I don’t feel like cooking, I will just go to the freezer”—00ML_15
3	“I have always struggled with my weight. And I’m older now. I have to try to eat right, so getting my protein, getting my fresh vegetables and I love my fresh fruit. I suffer with IBS. If you know what IBS is. So I need my fresh fruits and vegetables to help me with that. And everything is just getting so, so expensive that it’s ridiculous. I can’t imagine the struggle I would have if I didn’t have my food stamps. I don’t even wanna think about that because it’s difficult enough with my food stamps.”—00MP_49
4	**“**Oh my God. It [a SNAP decrease] would kill me. I mean, not right away, because like I said, with this extra money, that they’ve been giving me, I’ve been stocking up on a lot of my perishables and stuff like that, and my canned goods and stuff, and making sure I’m good to go. But it would really devastate me. I would have to go back to doing the pantry once a week and stuff, you know? And I do believe that the pantry is for the people that really need it. You know what I’m saying? But I’m like, since they’ve been giving me extra money, I’m not going to go and take away from the people that really, really need it. You know what I mean? So that’s why I stopped going once a week and I just started going once a month. To where I’m at now once a month. But if I was to get a decrease, now I’m sure that I would have to go back to once a week.”—00MP_18
5	“And that’s our government, and it’s even worse because I’ve worked for this and now that… I paid my taxes, I’ve paid my FICA and everything. I’m entitled to this. I’m not out here like some of these people who really don’t need it, or just having kids just to get it. But when you work all your life and then you lose your job and now you need some help from public assistance and you just have to go through all of that and then you have to repay stuff back, when you got these [explicit censored] that’s sitting down there collecting these benefits, and they walking in there with fur coats and gold diamonds and jewelry and all that stuff. And you just throwing money at ’em, as opposed to a hardworking man or a woman that’s worked all their life and they lost their job, and now they can’t even afford to feed their family”—00FB_11
6	“Because I don’t like to be a burden to people. And I feel like when I had to go to people that I know… I mean, there’s programs out there to help supplement most of whatever you need. So if I feel like I’m running low on groceries, instead of calling up a family member and asking them to buy me some groceries, I go to the food pantry. Now, if I go to the food pantry and I see it’s not time for my check and I’ve already been to the food pantry, my food stamps are out, yeah, then I will call family. But other than that, no, I’m gonna utilize all my options ’cause I don’t wanna be a bother to nobody. And we live in a world where we have help. So I utilize that first before I bug anybody. Well, I consider that bugging anybody.”—00MP_49

Abbreviation: FICA, Federal insurance contributions act; IBS, Irritable bowel syndrome.

More detailed planning, such as looking at flyers and navigating multiple stores, was more common among households experiencing food security compared with households at risk for experiencing food insecurity. Furthermore, households experiencing food security more commonly described buying food, especially meat, in bulk and breaking the larger quantity of food into smaller quantities for later use. Freezing both raw ingredients and leftovers was a common practice among many households, and specifically, all households experiencing food security, to stretch food and save money for other household expenses (Table 3, quote 2). These practices that promoted food security were described as time intensive by many people interviewed. Some people, particularly those at risk for experiencing food insecurity, described not having the luxury of time or cooking skills needed to be able to employ these strategies to get the best price for food or stretch food to last across multiple meals.

#### Domain 1, theme 2: the use of SNAP helps households meet their food needs, and the increase in benefits during COVID-19 promoted food security

All households that received SNAP (*n =* 34) in the past year shared how grateful they were for the benefit, and, of note, nearly all (*n =* 8) people who experienced food security used SNAP to buy food in the past year. Before the increase in benefits, people described their SNAP benefits as running out by the second or third week of the month. With the increase in benefits, nearly all people using SNAP shared that their benefits would last until the end of the month, with some people having leftover benefits rolling into the next month. Many households said the increase in benefits allowed them to buy foods they found to be more desirable and healthier, such as produce and meats (Table 3, quote 3). In addition, the few households that did use their SNAP benefits at farmers markets shared that the nutrition incentive program allowed them to buy even more fresh foods.

All households that used SNAP relied on SNAP to cover the majority, if not all, of their food costs in each month. If SNAP allotments ran out, most households described supplementing their food supply from a food pantry. Furthermore, the use of SNAP and the increased benefits allow for financial resources to become available for other household costs, such as car or home repairs. All people reflected that a SNAP decrease would cause financial hardship, but they would “figure it out.” Commonly “figuring it out” was described as cutting back on the amount of fresh foods, snack foods, and also stretching food available through different cooking strategies (for example, using less ground meat in pasta sauces). People also described needing to use charitable food sources more should they face a SNAP benefit decrease (Table 3, quote 4). In addition, nearly all households described how inflation made their food budgets tighter, despite having additional SNAP allotments. Households at risk for food insecurity described changing their shopping patterns because of inflation, with many specifically describing buying less meat and fewer eggs. All people shared they felt SNAP should remain, but that the program should be expanded to cover more populations, provide additional benefits permanently, and implement procedures that make accessing and enrolling in the program easier.

#### Domain 1, theme 3: nutrition equity is undermined by negative experiences enrolling in, retaining, and using SNAP

Despite the universal positive views and gratitude for SNAP, administrative burdens and unclear communication about the program were also universal. Most people described the SNAP application as being too long and needing too much information. People reflected positively on simplified and more accessible application and recertification procedures, such as online applications and completing eligibility interviews over the phone. People shared mixed emotions regarding communication from their state and local agencies about SNAP. Some people were happy with receiving communication only by mail, whereas others felt this was an outdated and inefficient way to receive updates. A few people shared that because of mail communication, they missed important recertification deadlines or notices about changes in benefits. Some felt communication by e-mail or phone would be better. Interestingly, despite the many changes made to SNAP during COVID-19, including benefit increases, no person interviewed who received SNAP could recall receiving any communication about these changes. Instead, all people described hearing about the changes through word-of-mouth or the news (for example, talking with their friends who also use SNAP).

Some people also shared experiences of disrespect and stigmatization in relation to the social benefit system. For example, a few people shared challenging experiences with caseworkers that made them feel stigmatized for applying for SNAP benefits, such as they were unworthy of receiving the benefit. A few respondents interviewed, all of whom received SNAP themselves, shared discriminatory remarks about certain populations receiving SNAP benefits, including people with “too many children,” people who experienced homelessness, and people who were not United States citizens (Table 3, quote 5). Although this was not common, these actions from people working in the social benefits system, and among people receiving social benefits, perpetuate social violence within the system.

#### Domain 1, theme 4: people navigate the complexities of the food system with resilience while trying to maintain independence from people outside their household

People commonly shared their desire to maintain their independence and the ability to meet their food needs without the support of people outside of their household (Table 3, quote 6). This, in part, was attributed to being unable to reciprocate support in many instances or knowing that people in their network were also struggling to meet their basic needs. If support was needed because of insufficient economic resources for food, people preferred to first use charitable food support and then would turn to family members or friends if additional support was needed. People most relied on family members for support as compared with friends or neighbors, especially for financial support or food sharing. People experiencing food security more commonly reported having people to turn to in times of need and being able to provide support to others. The most common support reported being received across all households was transportation support to and from food stores. If support was given to others, it was commonly sharing leftover food or sharing a meal.

### Domain 2: supply and demand for fresh and healthy foods

#### Domain 2, theme 1: acute and chronic violence (that is, interpersonal, and structural racism) against Black residents limits the supply of fresh foods despite the demand being present

All households shared feelings of grief when discussing the impacts of the deadly and racially motivated shooting at the only supermarket in the study area (Table 4, quote 1). Despite the physical site being associated with the traumatic memories of May 14, all people interviewed felt that the supermarket should reopen given it was the only supermarket within walking distance for many. People who primarily shopped at this supermarket shared that the massacre and temporary closing of the store made it challenging for them to get all the food they wanted and needed, especially produce in some cases.

**TABLE 4:** Illustrative quotes: supply and demand for fresh and healthy foods

Quotation number	Quotation
1	“You’re literally going there for a reason and one reason only. You don’t go to the grocery store and think about getting into a physical altercation or being met by a gun, by somebody’s or thinking about getting killed. It is, it’s very traumatic. It makes you feel like, now should I even step twice into the certain stores or going into certain neighborhoods into certain stores due to just people’s actions. So it’s something that you have to just pay attention to and be aware, but it’s very alarming, and it’s very hurtful. And it makes you think twice about certain things and about feeling like if you’re even welcome to come to this place, which is something that you shouldn’t think about at all. Because as people, I feel like we should be able to move how you want to move as far as being able to shop freely and do what you need to do. Like, we’re all at the grocery store to feed ourselves and our family”—00BF_09
2	“I just feel like in our community, they build like Chick-fil-A… Not Chick-fil-A. They build Checkers. They build like Checkers, and McDonalds, and Burger King, but you go down to the suburbs, they got core eating, and whole food eating, and good eating. All these things that are not in our community, but we want to eat and be healthy just like everybody else. So I just feel like if we need more healthy eating in the Buffalo community, in the east and the west side.”—00MP_45
3	**“**Oh my God. I sometimes, you know before I was getting extra food stamps. It was a challenge. I was going to the pantry more, and but now, like I said, now that I’m getting extra food stamps, I don’t have to go to the pantry as much. But I still go to keep that the food flowing, you know what I mean?”—00MP_18
4	“I used to go to the food pantry and they would give us some canned goods. Maybe you get a loaf of bread that’s about to go stale. Sometimes you might get lucky enough if you hit at the right time, we get some pastry. But other times it was a hit and miss. And especially even with meats, everything that you got in the meat department, it was tuna fish, it was chicken breasts in the can. Well, that’s all good, but sometimes, you want something other…’Cause I swear, if I went to daggone food pantries and they gave me one more can of tuna fish, I’m gonna throw it back at ’em”—00MP_49

All people described that it was challenging to find fresh and high-quality foods in their neighborhood and would travel further distances, such as to the suburbs, to get the food they preferred. Some people also described that the existing transportation infrastructure made it very challenging to get to other supermarkets outside of the study area if they did not have access to a vehicle. Others reflected on the disinvestment in the study area and overall food environment being different (for example, poorer quality foods in stores, more fast-food restaurants, more liquor stores) than other areas of the city that had higher area incomes and had a higher proportion of white residents (Table 4, quote 2). Reflections about food access in the study area did not differ between people experiencing food security and those being at risk for food insecurity.

#### Domain 2, theme 2: charitable food sources supply relief but do not typically promote nutrition equity as the supply of nutritious foods is limited

Many people interviewed described using charitable food sources, specifically food pantries, as a place they would get food (Table 4, quote 3). Both people experiencing food security and those at risk of food insecurity described using charitable food sources, but this was more common for people at risk of food insecurity. Some people regularly used food pantries (that is, weekly), whereas others used food pantries only when other resources for food had been depleted. All people who used food pantries felt the nutritional quality and freshness of the foods were poor, and often the foods provided were described as undesirable (Table 4, quote 4). These undesirable foods of poor nutritional quality were described as cereals, snack foods, canned meats, and canned soups; fresh produce, eggs, and milk were commonly described as desirable foods that were rarely available. Others shared that it was challenging to identify hours of operation at pantries, particularly during the pandemic, and wished delivery services were offered because of limited mobility. Use of other charitable food sources, such as meal sites or prepared meals, was rarely reported, but those who did use them shared similar views regarding the lack of nutritional quality and desirability of what was provided.

### Domain 3: community empowerment and food sovereignty

#### Domain 3, theme 1: community involvement through volunteerism and charity is present

All people interviewed shared a commitment to their neighborhood, emphasizing the strength present in the community that typically presented through acts of volunteerism and looking out for neighbors. Many people shared experiences helping neighbors meet food needs by bringing them food from food pantries or sharing leftover food, when able (Table 5, quote 1). People also shared receiving some support from neighbors, such as receiving a ride to the food store. Compared with food secure individuals, those at risk for food insecurity were more likely to be unable to give as much as they would like to the community because of limited resources (financial, time, or material). Some people shared that their commitment to giving back was so strong that they found a way to make it happen through working with local organizations. For example, 1 participant at risk for experiencing food insecurity shared she always volunteered time with a local organization to give turkeys away door to door during the winter holidays. Another participant at risk for experiencing food insecurity shared that he would go to a food pantry to bring food back not only for himself but for others in his apartment complex as well.

**TABLE 5:** Illustrative quotes: community empowerment and food sovereignty

Quotation number	Quotation
1	“I have a lot of friends. They call me, some may be hungry and some may need food, and I always pack ’em up nice little care package bags, and they’ll come get it. I might be poor, but like I said, someone call me and they’re hungry and they need it, I’ll give it to them.”—00MP_70
2	“I was just gonna mention too, they were giving Uber and Lyft rides because of the Tops after the Jefferson shooting. But then those codes expired at the end of May, so it’s like Uber and Lyft forgot about everybody. They haven’t issued new codes, I tried reaching out to the media, and Channel 4 was the only one that responded, and out of all these stations and the newspaper, and they looked into it a little bit and they said, “Well, Uber said they were only doing the one code, ” Even if Tops at Jefferson wasn’t opening till closer to August. And I don’t think he found anything out about Uber, so it’s kind of disappointing that they had expiration dates on those codes, but I guess it is what it is.”—00FB_12
3	“So I had to start buying the food out of my paychecks, so that made me have to work more and spend less time with my children. So they have to be home by themselves and raise themselves. So after a while I had to reduce my hours, even though I like to work there… It’s like you try and work more but then that leads to losing your benefits. And then that just makes it impossible to make ends meet” —00PW_46
4	“Make it more accessible, open up more places where you can get things at because if you’re not driving, if you don’t have transportation, then you out of luck. You can’t get to… Some of these folks can’t get around. So, if there were more available sites. Let’s put it that way. Pay more attention to what’s going on in the community instead of just waiting on somebody to… Get some advocates out here who can come… Who can go door to door and talk to the people in the community and find out well this is what we need. There’s so many other things that the government could be doing and hell, they do it for everybody else, but they don’t do it for this community”—00DG_42

#### Domain 3, theme 2: immediate response and relief are necessary in times of crises but not sufficient to promote nutrition equity

In response to the massacre, there was an influx of charitable food donations to the neighborhood, like what was seen at the beginning of the COVID-19 pandemic. Some people reflected positively about this influx of support, sharing they were grateful for how the community and organizations came together to provide immediate relief. Others shared frustrations about the emergency food response because it took a tragedy to get attention to the area after years of perpetual disinvestment in the community by city leadership. Others reflected negatively on the lack of sustainability built into the response, underscoring the lack of resiliency within the food system. For example, 1 person described using a subsidized rideshare program that was offered for a short amount of time after the massacre to get to grocery stores outside of the area (Table 5, quote 2). This person felt that a short-term solution like this should be sustained to address a root cause of food insecurity (that is, lack of transportation) that existed before the massacre and will persist without sustained systems changes.

#### Domain 3, theme 3: attention and action must be given to the root causes of food insecurity

Many people reflected on societal conditions that perpetuate disparities in income, wealth, and access to other resources needed to live a life with dignity. Some people shared that receiving a higher-paying job caused them to lose SNAP benefits, which created an additional financial hardship as the income gained did not make up for the benefits lost (Table 5, quote 3). Others reflected how the current high food prices were causing financial strain, and they had no choice but to adjust their shopping behavior. Beyond SNAP addressing immediate needs, some people shared that higher-paying jobs and access to economic opportunity would also contribute to improving food security and promoting nutrition equity in the community.

Many people shared a need for sustained structural investment in the community to change the food system so all could access the food they need. People described a lack of engagement and proactive action from city leaders in the pursuit of creating healthier physical (for example, access to healthy food) and social (for example, reduced crime) environments in the study area (Table 5, quote 4). Nearly all people interviewed described a lack of investment in the study area compared with other white, higher-income areas of the city. Some people specifically highlighted how a recent influx of reactive attention to the study area was already fading away within 2 mo of the massacre. Many people described the need for new services to be created as well to help those who were harder to serve through traditional models. For example, many people described needing to build a door-to-door outreach service for older adults and people with limited mobility so they could receive food deliveries from food pantries.

## Discussion

This qualitative study provides a nuanced description of how access and use of social infrastructures both promote and undermine the advancements of nutrition equity in a racialized urban community. Residents in Buffalo, NY, described how they seek to obtain food with dignity by budgeting, using cooking skills, and maintaining independence when navigating the food system through using SNAP and charitable food sources in place of support from family members and friends outside of the household. Experiences after the massacre on May 14 sharply highlighted the impacts of racialized violence on healthy food access despite demand for it and revealed shortfalls of the charitable food system in advancing nutrition equity. Conditions of poverty, challenges accessing and affording healthy food, and the inability to reciprocate support to others undermined nutrition equity despite the available social infrastructures. Finally, people explained the challenges and impacts of disempowerment and disinvestment at the household and community levels by describing the harms of low-wage work on one’s ability to meet household needs and the harms of temporary “fixes” to conditions that perpetuate nutrition inequity.

Our findings reveal how racialized structures and policies in the social and physical spaces of a community hinder the advancement of nutrition equity [5]. At the household level, experiences of disrespect and stigmatization in the social benefit system contributed to negative experiences when trying to receive SNAP benefits among a few study participants. This is consistent with literature describing the impacts of administrative burdens, specifically stigmatizing experiences and encounters, on health outcomes and the ability to receive public benefit programs for which people are eligible [41]. In addition, this finding is consistent with a recent cross-sectional study of 150 Black residents living in South Carolina where it was found that a higher frequency of experiences of racial discrimination was associated with an increased likelihood of food insecurity [42].

This evidence emphasizes the need to continue to dismantle racism and anti-Blackness in the different spaces people navigate when seeking to obtain the resources needed to meet food needs with dignity. At the community level, the temporary loss of the only supermarket in the study area as a result of a mass shooting magnified the pre-existing food inequities present east of Main Street in Buffalo, NY, that stem from historical and present-day white supremacy and structural racism [6,43]. Structural racism is defined as the ways society reinforces systems of power and oppression that differentially distribute access to opportunities and resources in ways that routinely advantage white populations compared with BIPOC populations [44]. In the study area, and across other urban communities in the United States, racialized policies and practices such as redlining, housing discrimination, and gentrification all are deeply entangled and perpetuate nutrition inequity and food insecurity by making healthy food inaccessible to BIPOC populations in urban communities [6]. For example, a recent study in the United States found that across urban Census tracts, those that were historically redlined and tracts that had a higher present-day proportion of BIPOC compared with white people had significantly lower access to healthy food [6]. This inequity was illustrated in reflections of our study participants who described the lack of access to fresh foods in their community despite the demand being present. Instead, people described an overabundance of fast-food outlets, corner stores, and liquor stores, which is consistent with literature focused on food insecurity and food environments in other urban areas [13,45,46].

The goal of advancing nutrition equity is to address the root causes of inequity and food insecurity by returning power to the community, investing in the supply of fresh and healthy foods, and creating means to navigate the local food environment with dignity [5]. Future research should test innovative cross-cutting solutions that seek to empower residents while simultaneously investing in the supply of healthy foods. One example of a cross-cutting solution includes the development of the Black Farmer Fund, which provides low-risk access to economic capital for Black growers and food business owners in the Northeast region of the United States to invest in the expansion of their farms or food enterprises [47]. Furthermore, progressive policy agendas that address the root causes of food insecurity, such as providing reparations or increasing minimum wages, could begin to address decades of disinvestment in BIPOC communities. Yet, the unintended consequences of these solutions should be closely evaluated and mitigated. For example, a recent qualitative evaluation of a minimum wage increase in Minneapolis found mixed results on health because there were overall minimum gains from the wage change that were paired with decreases in SNAP benefits because of increases in earned income [48].

Consistent with prior literature, people in this study also exhibited food resourcefulness and cooking knowledge to overcome barriers presented by social infrastructures [13,45,46,49]. Those experiencing food security, in particular, demonstrated food agency (an improved ability to acquire and prepare food to meet their household food needs) through their use of available resources (for example, food budgeting) and skills (for example, stretching leftovers) compared with people experiencing food insecurity [50]. This included carefully planning meals, knowing where to buy food for the best price, and putting considerable time and resources into planning how limited resources could be stretched to make ends meet, all of which are strategies reported in prior literature [49]. Findings from this USDA study suggest that compared with people experiencing food insecurity, those experiencing food security may have easier access to multiple different grocery stores, have support to draw from outside of the household, and may have more stable employment contributing to greater availability of time, transportation, and financial resources to navigate the complexities of the local food system [49]. In our study, we found that those experiencing food insecurity were less likely to have people outside of their household to turn to for support compared with those experiencing food security because of the inability to reciprocate support, experiences of poverty being common throughout networks, and not wanting to be a burden to others. These reasons are also consistent with prior literature seeking to understand how people experiencing food insecurity do and do not use their networks for support [23,49].

As highlighted by participants’ experiences, particularly after the temporary loss of the only supermarket in the community, advancing nutrition equity will require permanent, not temporary, investment into community infrastructures. One sector of the food system includes mutual aid organizations, which are defined as local, grassroot organizations that seek to provide resources to the community to promote stability in residents so they can reinvest back into their community [28]. Mutual aid organizations in the food system have historically addressed gaps not addressed by federal nutrition assistance programs and the different sectors of the charitable food system [28]. Unlike federal programs and many charitable food sources, mutual aid organizations work within the local food systems of farmers, producers, and consumers to not just provide support to those in need, but to also invest in and advance the grassroots infrastructure present [28]. Investing in the existing, community-led infrastructure is one way to foster community empowerment and food sovereignty, which is crucial for the advancement of nutrition equity [5,28]. In this study, participants were unable to identify sustained investments into the community (for example, generating wealth among community members), but many recommended mechanisms (for example, creating jobs for residents, improving wages, expanding social benefit programs) that promote economic development. Future community-based research and practice should develop models for sustained resource allocation to community-led organizations, such as mutual aid and grassroots organizations, to evaluate their potential for advancing nutrition equity and promoting food security among residents. For example, future work could assess the impact of expanding a local, Black-led food co-op in Buffalo, New York, on both wealth generation among BIPOC people in the city and the impact this expansion has on healthy food access [32].

This study has limitations to consider. First, the majority of the study sample identified as Black, low-income, female residents in an urban area with a mean age over 50 y; this limits the generalizability across other populations but is also a strength given this is a population that is most impacted by food insecurity and nutrition inequity in the United States. [1]. The findings contribute to an overall limited body of scholarship describing how people impacted most by food insecurity navigate their physical and social spaces to meet food needs in the pursuit of nutrition equity. Describing the experiences and lives of people included in this research provides evidence that can be translated into future programs, policies, and research priorities to address the root causes of persisting disparities in food insecurity. Second, we recruited people from a pool of participants that previously interacted with the research team through a household survey. This sampling strategy contributed to being able to build rapport quickly with participants given prior trust built in the previous engagement, but also likely contributed to limiting the perspectives collected and introduced selection bias to our study sample given we included people who were more willing and able to participate in research activities (that is, they reconsented to being contacted after the survey and were successfully reached upon recontact). Finally, to prevent response burden, we screened participants for the risk of food insecurity compared with the experience of food insecurity, rather than on a more precise spectrum (using more complex food security classification tools). Although this limited the burden on people involved in the research, it presented challenges in being able to capture nuances of experiences across the spectrum of food security.

In conclusion, this study provides evidence regarding how the availability and use of social infrastructures affect both food security and nutrition equity in a racialized urban community. The experiences documented through the major themes highlight the immense resilience, knowledge, and resourcefulness of individuals in conjunction with support from social infrastructures when seeking to meet food needs. Sustained and community-led investment into the local food system is needed to address structural inequities preventing the advancement of nutrition equity. In addition, easing administrative burdens and expanding social benefit programs to be more inclusive and supportive of low-income populations as they seek to generate wealth is necessary. Future participatory and action-oriented research is needed to identify pathways and solutions that leverage existing community strengths to advance nutrition equity in racialized urban food systems.

## Acknowledgments

We wish to acknowledge and honor the lives lost in our study area in Buffalo on May 14, 2022, as a result of a tragic mass shooting. May you rest in power. The study team wishes to acknowledge the Buffalo East Side Community who participated in this research for their willingness to share their stories and experiences.

## Author contributions

The authors’ responsibilities were as follows – GH, SG, AD, AM, PS, SR, YM: contributed to research design; GH, KA, YM: conducted research and analyzed the data; GH: wrote the article and had responsibility for the final content; and all authors: read and approved the final manuscript.

### Conflict of interest

The authors report no conflicts of interest.

### Funding

This work was funded by the Food & Agriculture Research Foundation (no grant number), with matching funding provided by Appetite for Change, Johns Hopkins University, Massachusetts Avenue Project, University at Buffalo, University of Minnesota, and Urban Fruits & Veggies. This was was also funded by the 21st Century Cities Initiative of the Johns Hopkins University. GH received funding through Procter & Gamble to support her doctoral studies (cost of living) during this research. Procter & Gamble had no role in the design, analysis, or writing of this article.

### Data availability

Data described in the manuscript, code book, and analytic code will be made available upon request pending application and approval by the research team (including academic and community investigators).

## Appendix A Supplementary data

Multimedia component1 Multimedia component1
