# Supplementary material for: “We Want to Eat and be Healthy just like Everybody Else:” How Social Infrastructures Affect Nutrition Equity in a Racialized Urban Community in the United States
Source: Curr Dev Nutr. 2024 Feb 21;8(3):102106. doi: 10.1016/j.cdnut.2024.102106 (PMC10937309; doi:10.1016/j.cdnut.2024.102106)
Supplement: Multimedia component1 [file mmc1.docx]

**Supplemental Table 1: Characteristics of study sample compared to eligible study sample selected from a previous household survey**

|  | **Study sample** | | **Eligible sample** | |  |  |
| --- | --- | --- | --- | --- | --- | --- |
| **Characteristic** | n=40 | 100% | n=111 | 100% |  |  |
| ***Food security status^a^*** |  |  |  |  |  |  |
| At risk for food insecurity | 30 | 75% |  |  |  |  |
| Not at risk for food insecurity | 10 | 25% |  |  |  |  |
| Food insecure |  |  | 57 | 51% |  |  |
| Food secure |  |  | 54 | 49% |  |  |
| ***Demographics*** |  |  |  |  |  |  |
| Race/ethnicity, n (%) |  |  |  |  |  |  |
| Black alone | 35 | 88% | 98 | 88% |  |  |
| White alone | 3 | 8% | 8 | 7% |  |  |
| Other race/ethnicity alone^b^ | 2 | 5% | 5 | 5% |  |  |
| Gender identity, n (%) |  |  |  |  |  |  |
| Female | 28 | 70% | 77 | 69% |  |  |
| Male | 12 | 30% | 33 | 30% |  |  |
| Refused to answer | 0 | 0% | 1 | 1% |  |  |
| Mean age, years (sd) | 53.3 | 12.4 | 51.4 | 13.5 |  |  |
| ***Socioeconomics*** |  |  |  |  |  |  |
| Household income, n (%) |  |  |  |  |  |  |
| <$15,000 | 19 | 48% | 53 | 48% |  |  |
| $15,000 - $24,999 | 15 | 38% | 27 | 24% |  |  |
| $25,000 - $34,999 | 4 | 10% | 16 | 14% |  |  |
| $35,000 - $49,999 | 2 | 5% | 7 | 6% |  |  |
| $50,000 - $74,999 | 0 | 0% | 3 | 3% |  |  |
| Refuse to answer | 0 | 0% | 5 | 5% |  |  |
| Homeownership, n (%) |  |  |  |  |  |  |
| Rent | 35 | 88% | 94 | 85% |  |  |
| Own | 5 | 13% | 17 | 15% |  |  |
| Vehicle ownership, n (%) |  |  |  |  |  |  |
| 0 vehicles | 23 | 58% | 57 | 51% |  |  |
| >1 vehicle | 17 | 43% | 54 | 49% |  |  |
| Education level, n (%) |  |  |  |  |  |  |
| <High school | 3 | 8% | 10 | 9% |  |  |
| High school graduate | 12 | 30% | 41 | 37% |  |  |
| Some college | 17 | 43% | 35 | 32% |  |  |
| College degree^c^ | 7 | 18% | 25 | 23% |  |  |
| Employment status, n (%) |  |  |  |  |  |  |
| Full time | 4 | 10% | 15 | 14% |  |  |
| Part time | 5 | 13% | 23 | 21% |  |  |
| Unemployed | 26 | 65% | 58 | 52% |  |  |
| Retired | 5 | 13% | 15 | 14% |  |  |
| Receipt of SNAP in the last 12 months, n (%) |  |  |  |  |  |  |
| Yes | 34 | 85% | 88 | 79% |  |  |
| No | 6 | 15% | 23 | 21% |  |  |
| Receipt of other social benefit programs, n (%)^d^ |  |  |  |  |  |  |
| Yes | 38 | 95% | 108 | 97% |  |  |
| No | 2 | 5% | 3 | 3% |  |  |
| Use of charitable food supports in the last 30 days, n (%)^e^ |  |  |  |  |  |  |
| Yes | 18 | 45% | 51 | 46% |  |  |
| No | 22 | 55% | 60 | 54% |  |  |
| ***Household composition*** |  |  |  |  |  |  |
| Marital status, n (%) |  |  |  |  |  |  |
| Married | 5 | 13% | 15 | 14% |  |  |
| Not married | 35 | 88% | 95 | 86% |  |  |
| Refused to answer | 0 | 0% | 1 | 1% |  |  |
| Average household size, no. (sd) | 2.5 | 2.0 | 3.0 | 1.9 |  |  |
| Any children in household, n (%) | 13 | 33% | 44 | 40% |  |  |
| Average number of children (n=13), no. (sd) | 2.5 | 2.1 | 2.0 | 1.5 |  |  |
| ^a^ Risk for food insecurity in the study sample was assessed at the time of eligibility screener using the two-item Hunger Vital Sign.^34^ Food security status was evaluated in the household survey using the USDA Six-Item Short Form Food Security Survey Module.^33^  ^b^ Includes Latinx, Asian, and Other race/ethnicity | | | | | | |
| ^c^ Includes Associate, Bachelors, and Graduate degrees | | | | | | |
| ^d^ Includes federal nutrition assistance programs, Farmers Market Incentives, Head Start, Temporary Assistance for Needing Families, Supplemental Security Income, Unemployment, Medicare, Medicaid, and Other Assistance | | | | | | |
| ^e^ Includes use of community food programs, food pantries, and faith-based organizations | | | | | | |

**Supplemental Table 2: Sample In-Depth Interview Questions**

| **Interview Component** | **Example Questions** |
| --- | --- |
| **Part 1: Food Acquisition and Spending** | 1. When deciding where to get food, what factors do you consider? 2. When deciding when to get your food, what factors do you consider? 3. When deciding what food to get, what factors do you consider? 4. In the places that you need to pay for food, what do you use to pay for food? 5. Can you describe what makes it easier to get the food you need? What makes it harder? 6. How do you cope during challenging times? 7. If your resources available for food fluctuate (for example, they become tighter), how does this impact your ability to get all the food you need? |
| **Part 2: SNAP** | 1. What are some things you like about SNAP? 2. What are some thing you wish were different about SNAP? 3. How, if at all, does SNAP fall short in meeting your household food needs? 4. Why do you no longer use SNAP? 5. Can you please walk me through the process of getting SNAP benefits? 6. How does the initial application process to receive SNAP benefits compare to the recertification process? 7. Can you describe to me the changes to SNAP you noticed during the pandemic (since March 2020) if any? 8. How did supplemental benefits provided during the pandemic impact your household? 9. If you could change anything about the SNAP program, what would it be? Why? |
| **Part 3: Social Support** | 1. How do people in your household impact your ability to meet your household food needs? 2. Some people’s family members or friends outside their household impact their ability to meet their household food needs. How do your family members or friends outside your household impact your ability to meet your household food needs? 3. Who helps you the most, if anyone? How do they help you? 4. How does the community around you impact how you get the food you need? 5. How, if at all, do you help people outside your household meet their food needs? 6. Can you describe the organizations or institutions in the community you use to get food? |
| **Part 4: Recommendations** | 1. My final question today is, what is needed for you to feel food secure (meaning you can get all the food you want and need for your household)? |
